# Retraction Note: Intracytoplasmic morphologically selected sperm injection versus conventional intracytoplasmic sperm injection: a randomized controlled trial

**DOI:** 10.1186/s12958-017-0279-9

**Published:** 2017-08-11

**Authors:** Giovanni Battista La Sala, Alessia Nicoli, Eleonora Fornaciari, Angela Falbo, Ilaria Rondini, Daria Morini, Barbara Valli, Maria Teresa Villani, Stefano Palomba

**Affiliations:** 1 Obstetrics and Gynecology Unit, Arcispedale S. Maria Nuova of Reggio Emilia, IRCCS, Reggio Emilia, Italy; 2 University of Modena and Reggio Emilia, Modena, Italy

## Retraction

The Editors-in-Chief are retracting this article [1] because the study was not conducted with the appropriate ethical oversight. Contrary to the statement in the article that ethics committee approval was not required, an investigation by the Arcispedale Santa Maria Nuova-IRCCS has determined that the study should have been reviewed and approved by an ethics committee. All authors agree with this retraction.

The online version of this article contains the full text of the retracted article as electronic supplementary material.
